# Melatonin mitigates root growth inhibition and carbon-nitrogen metabolism imbalance in apple rootstock M9T337 under high nitrogen stress

**DOI:** 10.3389/fpls.2024.1482351

**Published:** 2024-10-14

**Authors:** Maoxiang Sun, Chaoran Wang, Guowei Zhang, Hui Cao, Fen Wang, Ming Li, Shunfeng Ge

**Affiliations:** ^1^ Key Laboratory of Biochemistry and Molecular Biology in University of Shandong Province, School of Advanced Agricultural Sciences, Weifang University, Weifang, Shandong, China; ^2^ College of Agriculture & Forestry Technology, Weifang Vocational College, Weifang, Shandong, China; ^3^ Apple Technology Innovation Center of Shandong Province, Collaborative Innovation Center of Fruit & Vegetable Quality and Efficient Production of Shandong Province, College of Horticulture Science and Engineering, Shandong Agricultural University, Tai’an, Shandong, China

**Keywords:** apple rootstock, high N, melatonin, carbon-nitrogen metabolism, ^13^C, ^15^N

## Abstract

Nitrogen (N) is an essential element for plant growth, development, and metabolism. In apple production, the excessive use of N fertilizer may cause high N stress. Whether high N stress can be alleviated by regulating melatonin supply is unclear. The effects of melatonin on root morphology, antioxidant enzyme activity and ^13^C and ^15^N accumulation in apple rootstock M9T337 treated with high N were studied by soil culture. The results showed that correctly raising the melatonin supply level is helpful to root development of M9T337 rootstock under severe N stress. Compared with HN treatment, HN+MT treatment increased root and leaf growth by 11.38%, and 28.01%, respectively. Under high N conditions, appropriately increasing melatonin level can activate antioxidant enzyme activity, reduce lipid peroxidation in roots, protect root structural integrity, promote the transport of sorbitol and sucrose to roots, and promote further degradation and utilization of sorbitol and sucrose in roots, which is conducive to the accumulation of photosynthetic products, thereby reducing the inhibitory effect of high N treatment on root growth. Based on the above research results, we found that under high N stress, melatonin significantly promotes nitrate absorption, enhances N metabolism enzyme activity, and upregulates related gene expression, and regulate N uptake and utilization in the M9T337 rootstock. These results presented a fresh notion for improving N application and preserving carbon-nitrogen balance.

## 1 Introduction

Nitrogen (N) is an essential nutrient for plants (Krapp, 2015). It has multiple functions in plant metabolic processes and is pivotal for plant growth (Cunha et al., 2015; Mu and Chen, 2021). The application of N fertilizer can greatly increase grain yield (Abbaraju et al., 2022). As the potential for N fertilizer to increase yield is high and the price of N fertilizer is relatively low, farmers often overuse N fertilizer to maintain high yields (Wang et al., 2021). However, at present, the irrational application of N fertilizer leads to a low fertilizer utilization rate, and 60% of the fertilizer cannot be absorbed by crops, which increases the emissions of methane (CH_4_) and nitrous oxide (N_2_O) (Tian et al., 2020). It is reported that the world uses about 110 Tg (1 Tg = 1 million tons) of chemical fertilizer every year to meet the growing demand for food (Chen et al., 2020). In the past half a century, the total amount of N discharged into the environment by humans exceeded that of any other major element (Deng et al., 2024). Excessive N fertilizer input will not only cause air, soil, and water pollution, global climatic change, and ecological environment change, but it will also have a negative impact on crop growth, yield, and quality (Liu et al., 2021). This has serious implications for international food security and natural ecosystem stability. In fact, the excessive application of N fertilizer cannot fundamentally increase crop yield, because once the N fertilizer rate exceeds a certain threshold, the yield no longer increases significantly (Zhang et al., 2021). China is the world’s largest N fertilizer user, using more than 30% of pesticides and fertilizers on 9% of the world’s arable land (Sun et al., 2012). Plant growth stress caused by high N has become increasingly serious.

Although soils contain different forms of N sources, including NO_3_^-^, NO_2_^-^, NH_4_^+^and various organic N sources, most terrestrial plants, including apples, mainly absorb NO_3_^-^ (Feng et al., 2022). In addition to acting as a nutrient substance for plants, NO_3_^-^ can also act as a signaling substance to trigger local and systemic signaling pathways that regulate gene expression, metabolism, physiology, and plant growth and developmental processes, including seed germination, root construction, branch growth, and flowering time (Fredes et al., 2019). However, excessive NO_3_^-^ supply often has a negative impact on plant growth and development (Xu et al., 2016; Saiz-Fernández et al., 2015). It has demonstrated that excessive NO_3_^–^N reduces photosynthesis and enzyme activities, and increases ionic toxicity, osmotic stress and reactive oxygen species (ROS), further affecting crop quality and yields (Zhang et al., 2017). In previous studies, high concentrations of NO_3_^-^ in plants were considered a signal of N sufficiency to directly inhibit root elongation or indirectly affect root growth and development in crosstalk with phytohormones (Vega et al., 2019). However, it is well known that N metabolism is closely linked to other metabolic pathways, especially carbon metabolism. Wang et al. also reported that high N levels increased the synthesis of amino acids and their derivatives in apple fruit, and inhibited carbohydrate accumulation (Wang et al., 2022). Therefore, excessive N supply often causes imbalance of carbon and N metabolism and affects plant growth.

As a multifunctional molecule, melatonin is present in nearly all living organism (Antoniou et al., 2017). It is now widely accepted as a new endogenous plant hormone having pleiotropic activities in trace amounts (Ding et al., 2017). Melatonin is generally produced in the chloroplast and mitochondria of roots and/or leaves, and further transported to the meristem, flowers and fruits in plants (Wang et al., 2016). Evidence have been found that melatonin has several physiological functions in regulating root development, seed germination, photosynthesis, flowering, leaf senescence, and fruit maturation (Back et al., 2016; Arnao and Hernández-Ruiz, 2019). The antioxidant properties of melatonin can significantly reduce plant damage due to environmental stress at specific concentrations (Gomes et al., 2014; Wei et al., 2015). For example, one study found that exogenous melatonin application improved photosynthetic performance, reduced leaf hydrogen peroxide accumulation, and alleviated plant growth inhibition due to drought in *Malus hupehensis* by promoting the absorption of nutrient elements (Liang et al., 2018). Du et al. (2022) found that melatonin can relieve nutrient stress caused by low concentrations of NH_4_^+^ through regulating the absorption and metabolism of N. Recently, the roles of melatonin in nutrient uptake has become a research focus, especially in terms of N absorption and utilization (Liu et al., 2020).

The apple industry is an important agricultural industry in China. In recent years, China’s apple cultivation area has stabilized, and the output has increased steadily, China has become the largest apple-producing country in the world (Faostat, 2018). However, the site conditions of most apple orchards are poor, as they are mainly distributed on hillsides, hills, and beaches with relatively poor soil nutrient levels and low soil organic matter (Ge et al., 2018). Given the poor soil conditions of apple orchards, chemical fertilizer plays an important role in increasing yields. The pursuit for high yield has led to the sustained and rapid growth of N fertilizer application in Chinese apple orchards, which has far exceeded the demand of the trees (Zhu et al., 2018).

There is currently a lack of research on how melatonin regulates carbon-nitrogen metabolism in apple rootstock M9T337 under high N stress. In this study, we used apple rootstocks M9T337 as test materials to explore the effects of melatonin on the roots and the photosynthetic carbon metabolism of rootstocks under high N conditions. Our results should provide a basis for the rational application of N fertilizer.

## 2 Materials and methods

### 2.1 Experimental site and materials

The pot experiment was carried out between April and July 2022 at the Agricultural Science Experimental Station of Weifang University (118*°*10’ E and 35*°*41’ N). The soil utilized in the experiment was loam, with 22.13 grams of organic matter per kilogram, 46.81 milligrams of N per kilogram, 33.25 milligrams of accessible phosphorus per kilogram, and 178.12 milligrams of potassium per kilogram. The test materials consisted of *Malus domestica* M9T337 dwarf rootstocks. Rootstocks were cultivated in a soil culture system within a growth chamber, exposed to natural light. The growth chamber maintained a temperature cycle of 28°C during the day and 18°C during the night, as well as a humidity level of 65%. On April 9th, when the seedlings reached 10 true leaves, they were moved into pots. The pots had a height of 17 cm and a diameter of 22 cm, and contained 2.5 kg of soil each and each treatment was repeated in 30 pots (replicates). Only one plant was placed in each pot. After a period of 14 days, rootstocks exhibiting consistent development were chosen for the purpose of conducting experiments (about 13 cm tall).

In the formal trial, Ca(NO_3_)_2_ was used as the only N source. The seedlings were treated for root application as follows: CK (Control, 10 mmol·L^−1^ NO_3_^−^-N), 100 μmol·L^−1^ melatonin (MT, Kangwei Century Technology Co., Ltd.), the experimental concentration is referred to the study of Liang et al (Liang et al., 2018), 30 mmol·L^−1^ NO_3_^−^-N (HN), the experimental concentration is referred to the study of Liu et al (Liu et al., 2022), 30 mmol·L^−1^ NO_3_^−^-N + 100 μmol·L^−1^ melatonin (HN+MT). For the ^15^N labeling of plants, we added 0.5 g (CK and MT) and 1.5 g (HN and HN+MT) Ca(^15^NO_3_)_2_ (10.22% abundance, Shanghai Yuan ye Biotechnology Co., Ltd.) to each pots, each pots was treated with 200ml, once every 2 days, for a total of three times. After 30 days of treatment, samples were obtained to determine different indicators.

### 2.2 Determination of root morphology and root activity

The cleaned root systems that float on 5 mm of water in a 0.3 × 0.2 m Plexiglas tray. Roots be untangled and discrete with a brush to lessen root interlock. Greyscale root images got by tuning the parameters to ‘‘high’’ setting (resolution 300 by 300 dpi). Then WinRhizo software (WinRHIZO version 2012b, Regent Instruments Canada, Montreal, Canada) was using to analyze the whole root system (root length, Number of root tips, root surface area and root volume).

The root activity was determined by the 2,3,5-triphenyltetrazolium chloride (TTC) reduction method and expressed as the amount of TTC reduced by per gram of root (Gao et al., 2023).

### 2.3 Determination of biomass

The plant samples were washed and dried off with an absorbent paper. Then, the fresh weights of the aboveground and belowground parts were measured. The dry weights of the aboveground and below-ground parts were measured after drying the samples in an oven at 105°C for 30 min and at 80°C to constant weight (Sun et al., 2022).

### 2.4 Determination of antioxidant enzyme activities and H_2_O_2_

The superoxide dismutase (SOD) activity was determined based on the ability to inhibit the photochemical reduction of nitroblue tetrazolium (NBT) (Meftahizadeh et al., 2023). The peroxidase (POD) activity was determined by a colorimetric method in a reaction mixture containing guaiacol as the substrate (Wu et al., 2023). The POD activity was determined based on the change in absorbance at 470 nm due to the oxidation of guaiacol to tetraguaiacol. The supernatant was mixed with and was allowed to run. The absorbance of the reaction mixture was read at 560 nm. The catalase (CAT) activity was determined by the ultraviolet spectrophotometric method (Ren et al., 2022) at 240 nm in a reaction mixture. The CAT activity was measured based on the decomposition of H_2_O_2_ with time. Ascorbate peroxidase (APX) activity was measured by a spectrophotometric method according to Sun et al (Sun et al., 2022).

The H_2_O_2_ was determined according to the methods described by Yan et al (Yan et al., 2023). Pre-cooled acetone was used to extract the H_2_O_2_ in the sample and detected by monitoring the absorbance of the titanium peroxide complex at 412 nm.

### 2.5 Determination of soluble sugar content

Sorbose (Sor), sucrose (Suc), glucose (Glu) and fructose (Fru) were performed as described by Aslam et al. (2019). The hexose phosphates glucose 6-phosphate (G6P) and fructose 6-phosphate (F6P) were extracted and assayed as described by Chen et al. (2023).

### 2.6 ^13^C, ^15^N labeling method and isotope analysis

After 27 days of treatment, ^13^C isotope labeling was performed. Ba^13^CO_3_ (^13^C independence is 98%) was used as a selection marker, and the dosage was 0.2 g. The seedlings were placed together with the markers, fans, and reduced iron powder into a sealed marking room to make transparent film. The transmittance of sunlight in the labeling chamber was 95% of the natural light intensity. Labeling work started at 9:00 a.m., at which point the fan was turned on and the labeling chamber was sealed. One milliliter of hydrochloric acid (1 mol·L^−1^) was injected into the beaker with a syringe every 0.5 h in order to maintain the concentration of CO_2_, and the ^13^C labeling lasted for 4 h. In order to keep low temperature during the labeling process, an appropriate amount of ice was added to the bottom of the labeling chamber to limit the temperature within the range of 28–37°C. At the same time, three other plants were selected as the control (^13^C natural abundance), and destructive samples were taken on the third day after labeling and ^13^C was determined (Xu et al., 2020).

Biomass samples for ^13^C and ^15^N analyses were collected at the end of the labeling for ^13^C and ^15^N analyses. The samples were dried and then ground with an electric grinder and filtered with a 0.25 mm mesh screen. The abundance of ^15^N and ^13^C were measured with a ZHT-03 mass spectrometer from the Beijing Analytical Instrument Factory (Chinese Academy of Agricultural Sciences). Three replicates were conducted for each treatment. The formula is calculated according to Wang et al (Wang et al., 2020).

### 2.7 Measurement of NO_3_^−^ flux and N-metabolizing enzymes activity

The NO_3_^−^ flux at root surface were measured using a scanning non-invasive micro-test technique system (NMT 100 Series, United States) by Xuyue Sci. & Tech. Co., Ltd., Beijing, China. Net fluxes of NO_3_^−^. Each selected root was tested for 10 min, with 5 replicates per group. After the test, we analyzed the data with MageFlux (imFluxes V2.0; YoungerUSA LLC, Amherst, MA 01002, USA), a data analysis software provided by Xuyue company (Zheng et al., 2023).

The ammonium-nitrogen (NH_4_^+^-N) concentration was quantified using Nessler’s spectrophotometric technique (Molins-Legua et al., 2006). The leaf extract was combined with hypochlorite and phenol in a very alkaline environment to produce a water-soluble blue indophenol, with its absorbance measured at 625 nm. The activity of nitrate reductase (NR) in the leaves was assessed using sulfanilamide colorimetry (Zhao and Cang, 2015). The leaf extract was combined with sulfanilamide in hydrochloric acid and N-1-naphtyl-ethylenediamine to produce a red azo dye, with absorbance measured at 520 nm. Glutamine synthetase (GS) activity was assessed using the plant GS enzyme activity assay kit, and glutamic acid synthetase (GOGAT) activity was evaluated using the plant GOGAT enzyme activity assay kit (Jiangsu Enzyme Label Biotechnology Co., Ltd, Jiangsu, China), in accordance with the manufacturer’s instructions (Sun et al., 2022).

### 2.8 DNA extraction and quantitative PCR of genes related to carbon and N metabolism

Genes involved in N assimilation such as nitrate reductase (*MdNR*), glutamine synthetase 1 (*MdGS1*), and nicotinamide adenine dinucleotide-glutamate synthase (*MdNADH-GOGAT*); nitrate transporters such as nitrate transporter 1.1/1.2/1.5/2.1 (*MdNRT1.1/1.2/1.5/2.1*); Sucrose synthase (*MdSUSY1*); sucrose transporter (MdSUT1); Phosphosucrose synthase (*MdSPS1*) and hexokinase (*MdHK6*) were selected for transcript analysis by quantitative real-time PCR (qRT-PCR).

Total RNA was extracted using an RNAprep Pure Plant Kit (Tiangen, Beijing, China) according to the manufacturer’s instructions. The RNA was reverse-transcribed into cDNA using a RevertAid First Strand cDNA Synthesis Kit (TransGen) in a 20 mL reaction. The quantitative real-time PCR (qRT-PCR) was performed in a 20 mL reaction mixture containing 10 mL of Green qPCR SuperMix, 1 mL of cDNA, 2 mL (1 mL of upstream and 1 mL of downstream primers) of primers and 7 mL of ddH_2_O. The relative gene expression levels were calculated by the 2eDDCT method (Lv et al., 2012), and the MdActin gene was used as the internal control. These qRT-PCR experiments were performed with three technical replicates and three biological replicates. The primers used for qRT-PCR were listed in **Supplementary Table S1**.

### 2.9 Data analysis

The data presented as means (± SD) of three replicates. The data were subjected to one-way ANOVA and the least significant difference test (Duncan’s new multiple range method, p ≤ 0.05) using SPSS 20.0 software (Statistics software, version 20.0, IBM, USA). Significant differences were determined at a probability level of P ≤ 0.05.

## 3 Results

### 3.1 Effects of different treatments on the growth of M9T337 rootstock

**Figure 1:**
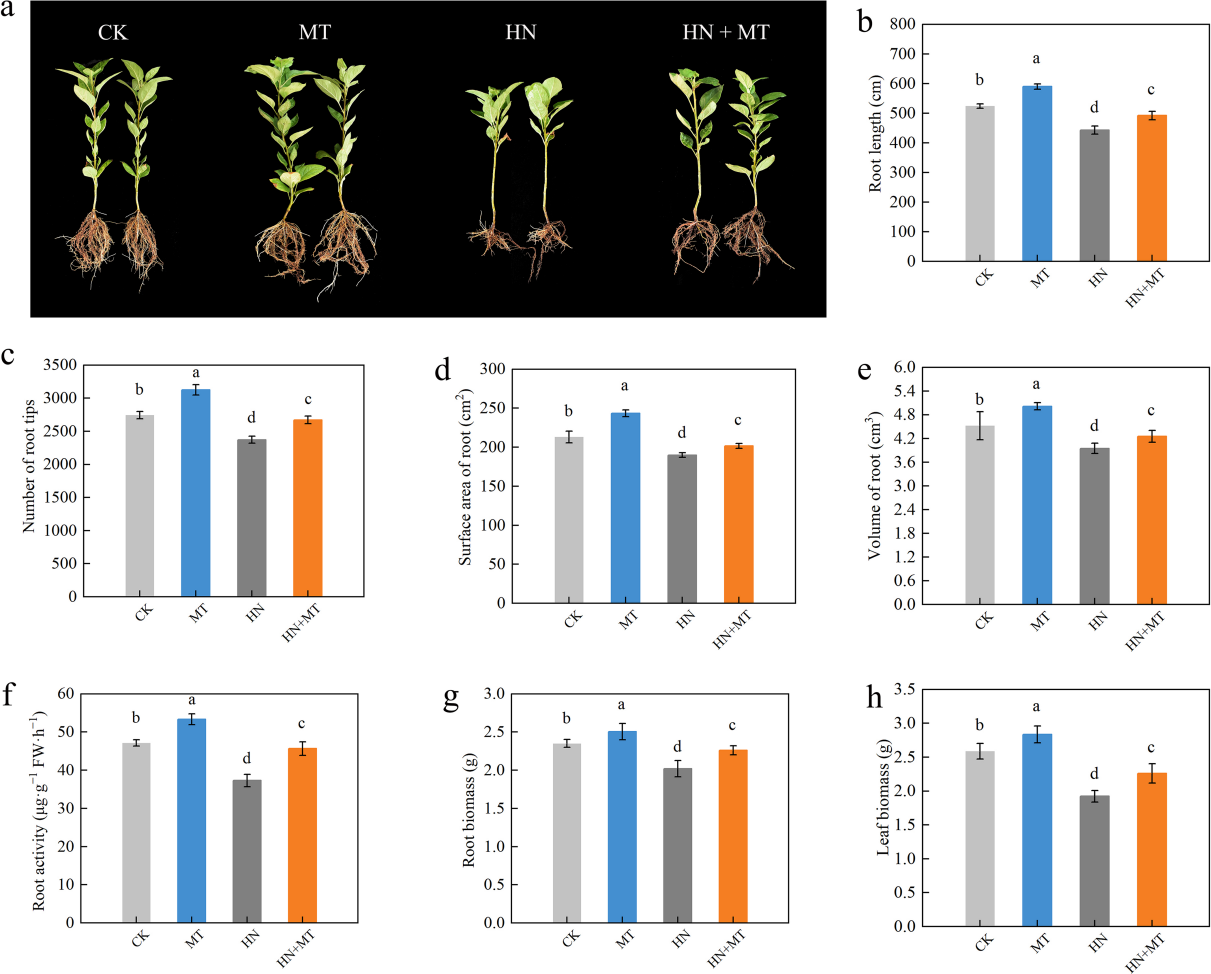
Effects of different treatments on the growth of M9T337 rootstocks. **(A)** Root growth. **(B)** Root length. **(C)** The number of root tips. **(D)** Surface area of the root. **(E)** Volume of the root. **(F)** Root activity. **(G)** Root biomass. **(H)** Leaf biomass. The error bar represents the standard deviation of the mean (n=3). Different lowercase letters indicate significant differences among treatments (Duncan test, *p* < 0.05).

Compared to CK, HN significantly inhibited the root growth of seedlings (**Figure 1A**). It was found that the MT treatment greatly improved the root morphology of plants that were given CK, and the HN+MT treatment greatly improved the effects of high N on plant root growth. For example, the root length, surface area, volume, and number of root tips were 14.79%, 27.29%, 46.53%, and 39.16% higher in the HN+MT treatment than in the HN treatment (**Figures 1B–E**). The root activity also showed the same trend (**Figure 1F**). **Figure 1A** shows that different treatments have different effects on M9T337 rootstock growth. The HN treatment can greatly lower the plant’s biomass in its roots and leaves. On the other hand, the HN+MT treatment can greatly improve M9T337 rootstock growth, which was slowed down by the high N treatment. Compared with HN treatment, HN+MT treatment increased root and leaf growth by 11.38% and 28.01%, respectively (**Figures 1G, H**).

### 3.2 Effects of different treatments on photosynthetic parameters and antioxidant enzyme activities

**Figure 2:**
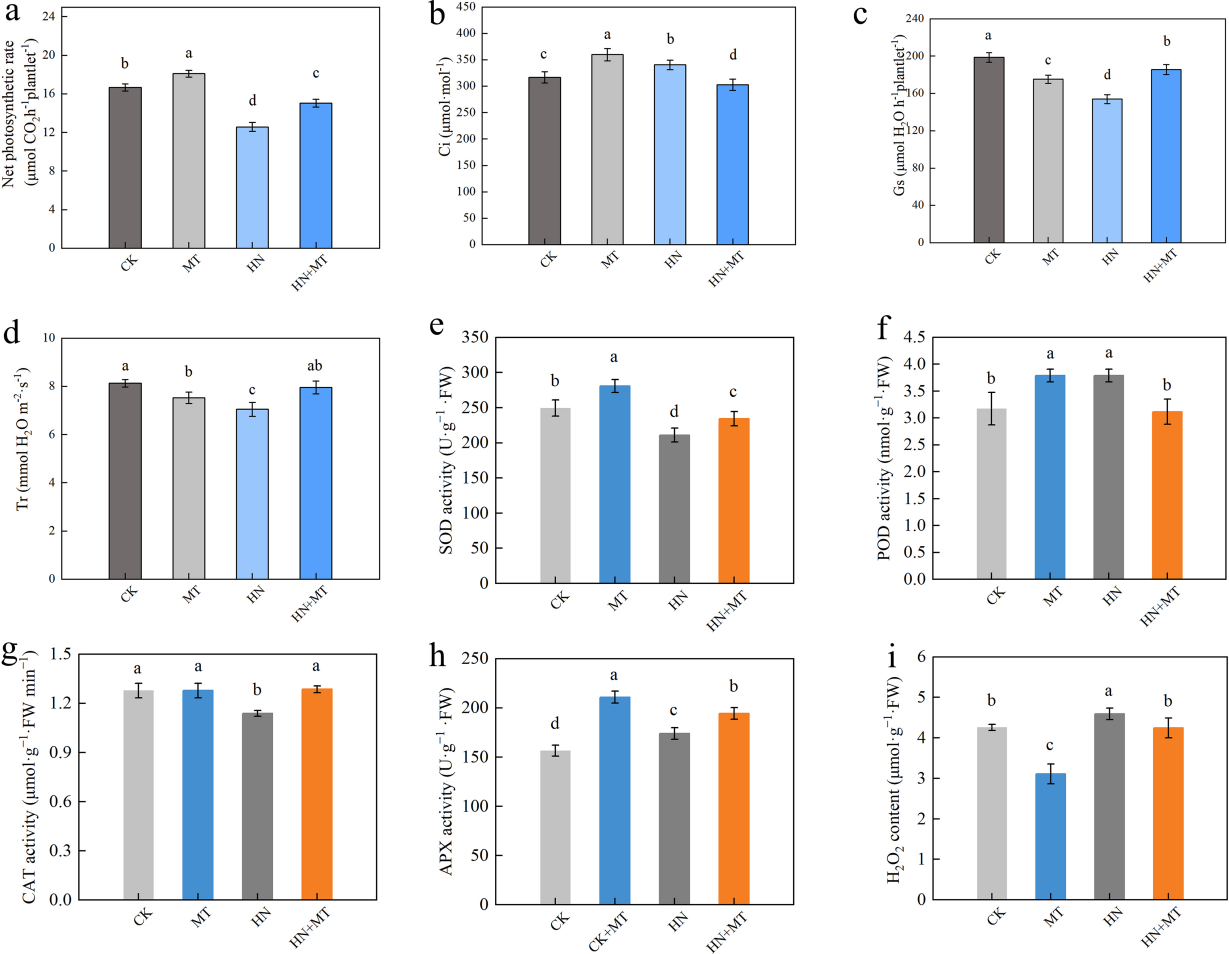
Effects of different treatments on photosynthetic parameter and the antioxidant enzyme activities in leaf of M9T337 rootstocks. **(A)** Net photosynthetic rate. **(B)** The intercellular CO_2_ concentration (Ci). **(C)** Stomatal conductance(Gs). **(D)** Transpiration rate (Tr). **(E)** SOD activity. **(F)** POD activity. **(G)** CAT activity. **(H)** APX activity. **(I)** H_2_O_2_ content. The error bar represents the standard deviation of the mean (n=3). Different lowercase letters indicate significant differences among treatments (Duncan test, *p* < 0.05).

Compared to CK, MT treatment increases the net photosynthetic rate of M9T337 rootstocks, HN treatment reduces the net photosynthetic rate of M9T337, while high N treatment with the addition of melatonin can significantly reduce the decrease in the net photosynthesis rate caused by high N. The HN+MT treatment of M9T337 rootstocks increased the net photosynthetic rate by 19.65% compared to the HN treatment (**Figure 2A**). MT and HN treatment significantly increased the intercellular CO_2_ concentration (Ci) of M9T337 rootstocks and reduced the stomatal conductance (Gs) and transpiration rate (Tr) compared to the control. Compared to HN treatment, HN+MT treatment significantly reduces the intercellular CO_2_ concentration of M9T337 rootstocks and improves the stomatal conductance and transpiration rate. Intercellular CO_2_ concentration decreased by 12.94% compared to HN treatment, and stomatal conductance and transpiration rates increased by 18.08% and 11.20%, respectively (**Figures 2B–D**).

MT treatment can significantly increase SOD activity, POD activity, and APX activity, contrary to HN treatment. Compared with CK treatment, SOD activity, POD activity, and APX activity of MT treatment were 12.56%, 19.45%, and 34.70% higher, respectively. However, compared with HN treatment, HN+MT treatment can significantly increase SOD activity, CAT activity, and APX activity under high N stress. Compared to HN treatment, HN+MT treatment had higher SOD activity, CAT activity, and APX activity (11.05%, 12.90%, and 5.92%, respectively) (**Figures 2E–I**).

### 3.3 Effect of different treatments on soluble sugar of M9T337 rootstocks

**Figure 3:**
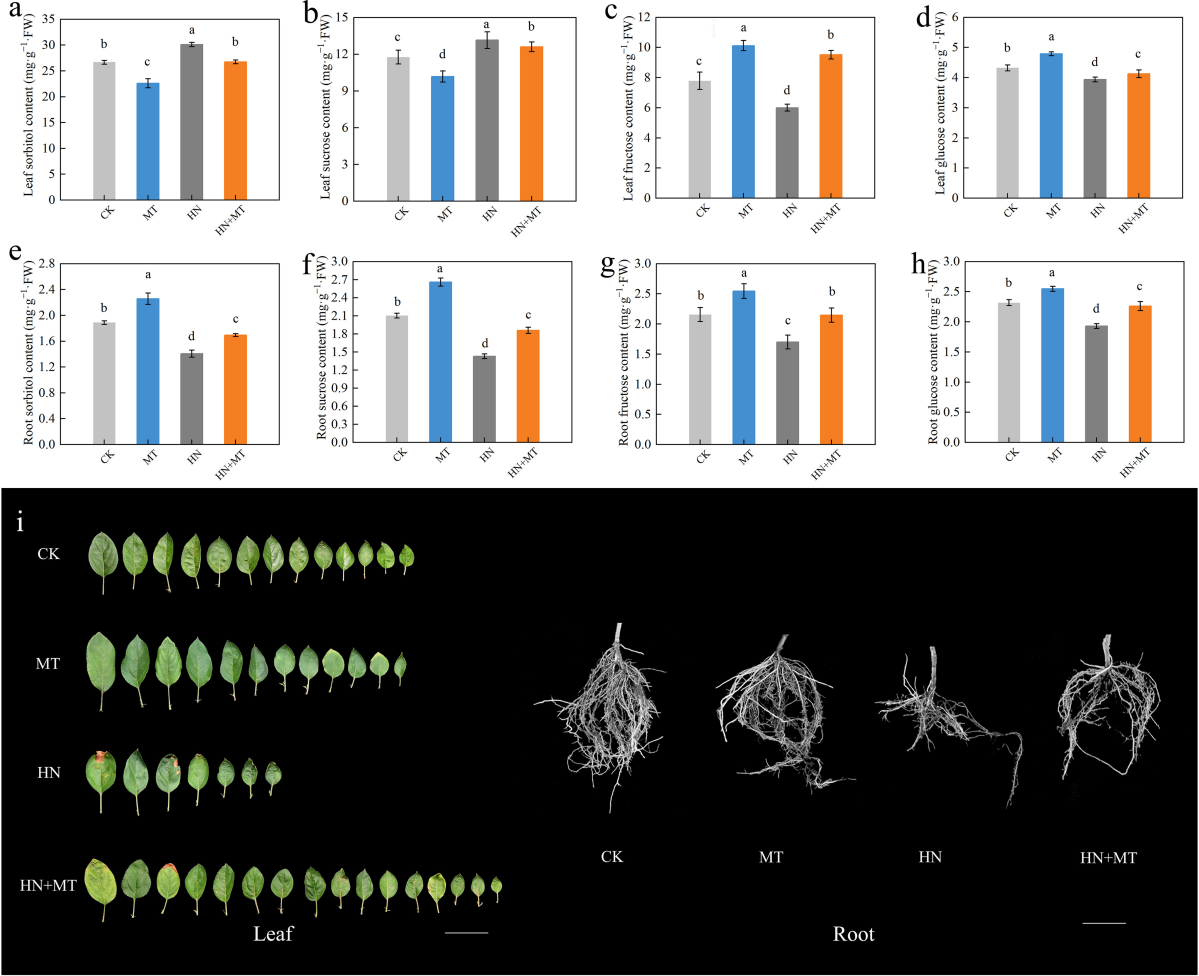
Effect of different treatments on the soluble sugar in leaf and root of M9T337 rootstocks. **(A)** Leaf sorbitol content. **(B)** Leaf sucrose content. **(C)** Leaf fructose content. **(D)** Leaf glucose content. **(E)** Root sorbitol content. **(F)** Root sucrose content. **(G)** Root fructose content. **(H)** Root glucose content. **(I)** Photo of root and leaf. The error bar represents the standard deviation of the mean (n=3). Different lowercase letters indicate significant differences among treatments (Duncan test, *p* < 0.05).

The soluble sugar content in rootstock leaves and roots under different treatments were determined (**Figure 3**). The content of sorbitol and sucrose in leaves under HN+MT treatment was lower than that under HN treatment and higher than that under MT treatment, while the content of sorbitol and sucrose in leaves under HN+MT treatment was higher than that under HN treatment and lower than that under MT treatment. Compared to the MT treatment, the contents of sorbitol and sucrose in leaves treated with HN+MT increased by 18.18% and 23.77%, respectively. In roots treated with HN+MT, the content of sorbitol and sucrose increased by 20.71% and 30.91%, respectively, compared to HN treatment (**Figures 3A, B, E, F**).

Leaves and roots treated with HN+MT had significantly higher fructose and glucose contents than those treated with HN. Compared with HN treatment, the fructose and glucose contents in leaves under HN+MT treatment were increased by 58.33% and 4.83%, respectively, and the fructose and glucose contents in roots were increased by 28.88% and 17.19%, respectively (**Figures 3C, D, G, H**).

### 3.4 Effect of different treatments on N metabolism of M9T337 rootstocks

**Figure 4:**
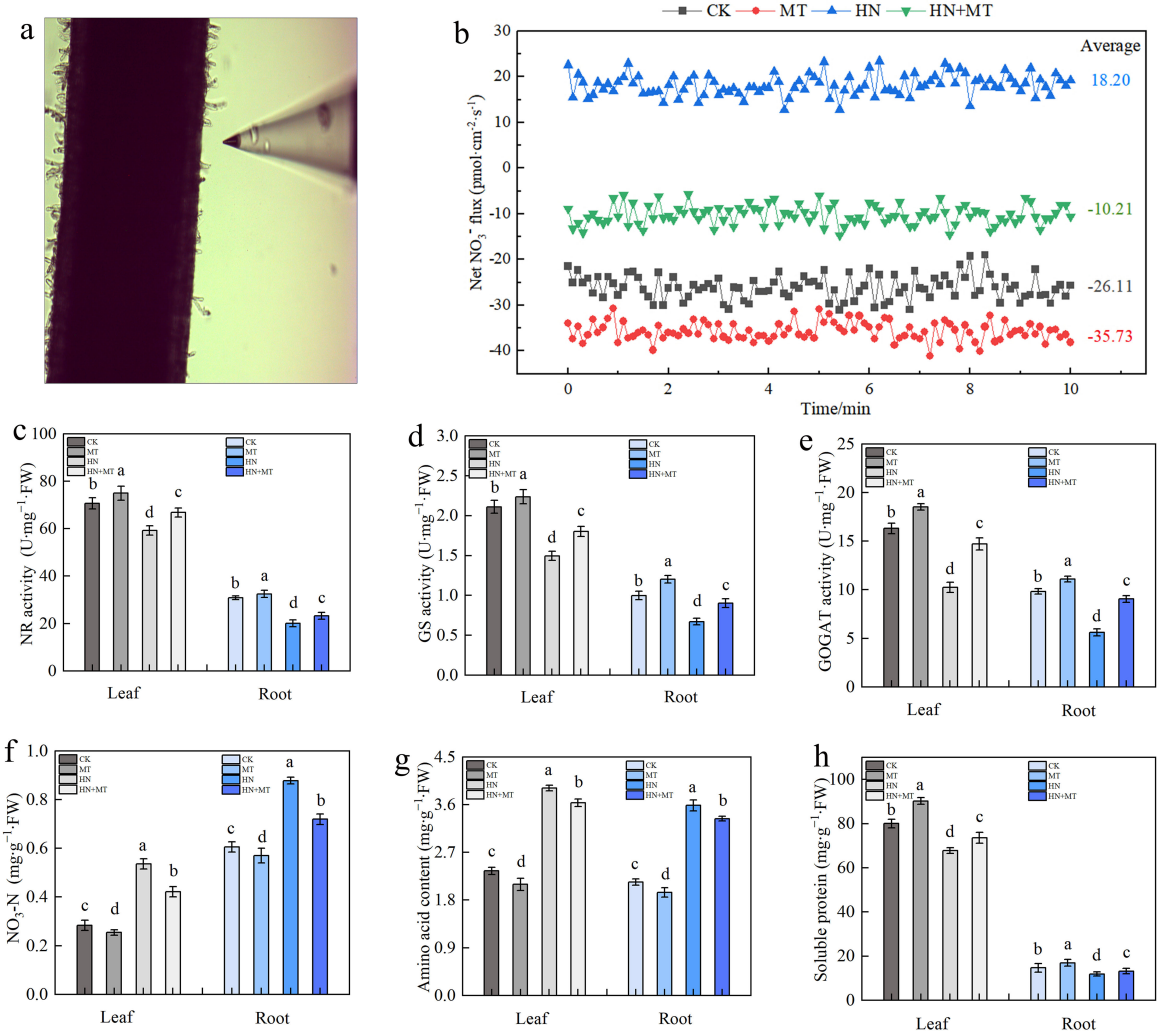
Effect of different treatment on N metabolism in leaf and root of M9T337 rootstocks. **(A)** NO_3_^–^ fluxes detection of roots. **(B)** Net NO_3_^–^ fluxes in the root of M9T337 rootstocks for 10 min. **(C)** NR activity. **(D)** GS activity. **(E)** GOGAT activity. **(F)** Nitrate nitrogen. **(G)** Amino acid content. **(H)** Soluble protein. The error bar represents the standard deviation of the mean (n=3). Different lowercase letters indicate significant differences among treatments (Duncan test, *p* < 0.05).

The rhizosphere NO_3_^–^ velocity of M9T337 rootstocks was significantly different under different treatments (**Figures 4A, B**). Negative values indicate NO_3_^–^ influx, i.e., NO_3_^–^ absorption, and positive values indicate efflux. Compared to CK, MT treatment promoted NO_3_^–^ absorption, with the average value changing from -26.11 pmol·cm^–2^·s^–1^ to -35.73 pmol·cm^–2^·s^–1^. In HN treatment, NO_3_^–^ is effluxed by roots, while in HN+MT treatment, NO_3_^–^ flow in rhiza is influxed, with an average rate of around -10.21 pmol·cm^–2^·s^–1^. The results showed that high N treatment was not conducive for NO_3_^–^ absorption, while high N treatment after adding melatonin is favorable to NO_3_^–^ absorption.

N-metabolizing enzymes play important roles in the process of N assimilation. Under different treatments, the activity trend of NR, GS, and GOGAT in leaves and roots remained the same. Compared to CK, MT treatment increased the activities of NR, GS, and GOGAT, while HN treatment inhibited these three enzymes. MT+HN treatment could reduce the inhibition of NR, GS, and GOGAT activities in high N treatment. Compared to HN treatment, NR, GS, and GOGAT in leaves increased by 12.84%, 20.49%, and 43.92%, respectively, while NR, GS, and GOGAT in roots increased by 12.84%, 34.44%, and 61.54%, respectively (**Figures 4C–E**). According to **Figures 4F–H**, it can be found that, compared with CK, the nitrate N and amino acid contents of leaves and roots under HN treatment significantly increased, while the soluble protein contents decreased. Compared with HN treatment, MT+HN treatment increased soluble protein content.

### 3.5 Transcriptional regulation of genes involved in carbon and N metabolism

**Figure 5:**
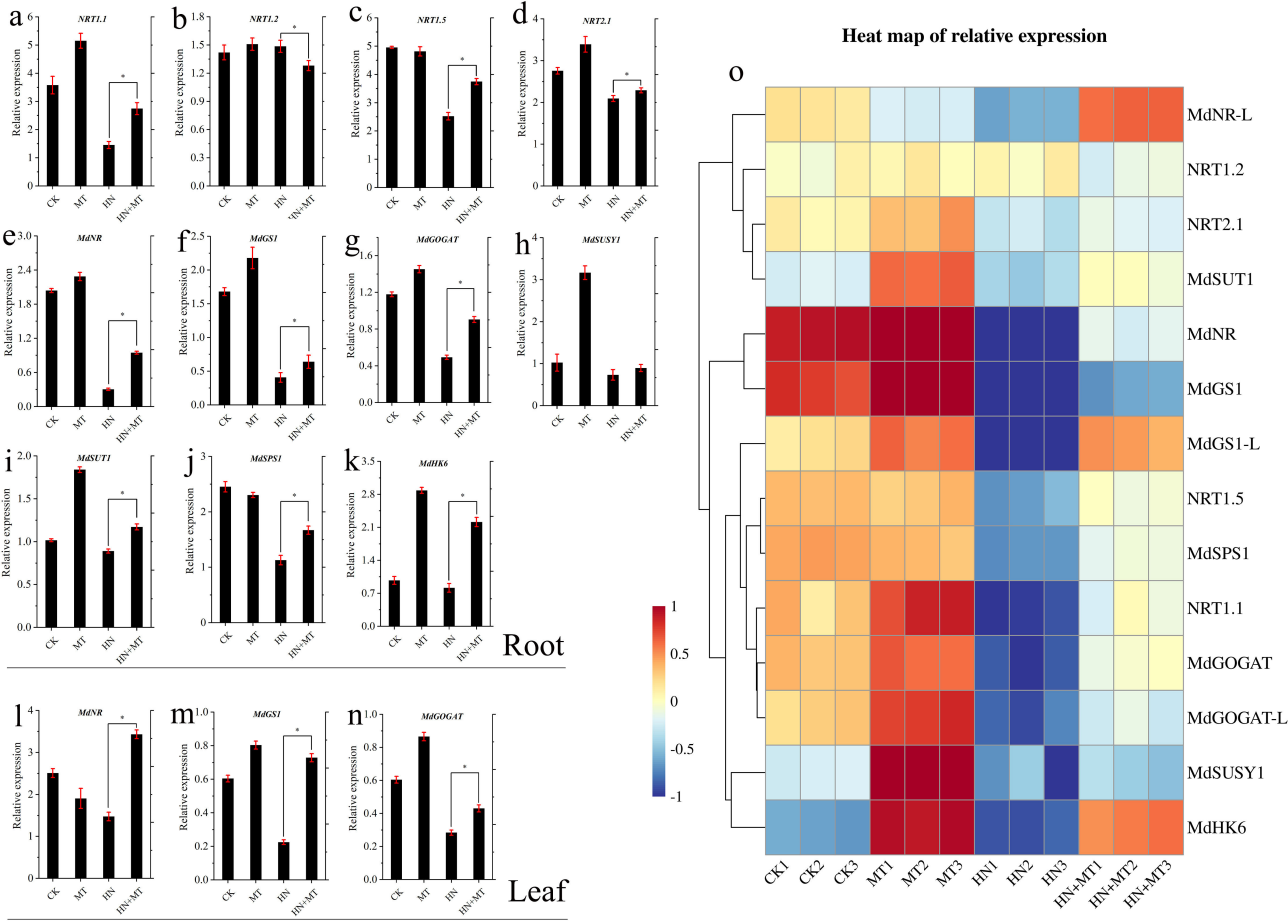
Effect of different treatment on transcriptional regulation of genes involved in carbon and N metabolism of apple rootstock M9T337. **(A–N)** The expression changes of carbon and N metabolism genes in root and leaf. (Duncan test, *p ≤ 0.05) **(O)** Heat map of relative expression. Color scale represents relative intensity of gene expression. The qRT-PCR results were standardized by Z-score and the data represents the Z-score of gene expression. The red color represents upregulated expression, while blue represents downregulated expression.

The expression changes of the N transporter gene and the N channel protein gene in the root and leaf systems are shown in **Figures 5A–N**. The transcription levels of *NRT1.1*, *NRT1.2*, and *NRT2.1* in the roots of M9T337 stock treated by HN were significantly lower than those treated by HN+MT. Under MT treatment, the expression levels of *MdNR*, *MdGS1*, *MdGOGAT*, *MdSUSY1*, *MdSUT1*, and *MdHK6* in the root were the highest. These results indicated that high N significantly inhibited the expression of the N transporter gene and the N channel protein gene. Compared with HN treatment, the expression of the N transporter gene and the N channel protein gene in the root and leaf was significantly up-regulated by high N treatment with melatonin.

According to the heat map of relative expression analysis in **Figure 5O**, the expression of the N transporter gene and the N channel protein gene in roots and leaves was significantly down-regulated under HN treatment, while HN+MT treatment could significantly up-regulate the expression of the N transporter gene and the N channel protein gene in roots and leaves.

### 3.6 Effect of different treatments on ^13^C and ^15^N accumulation of M9T337 rootstocks

**Figure 6:**
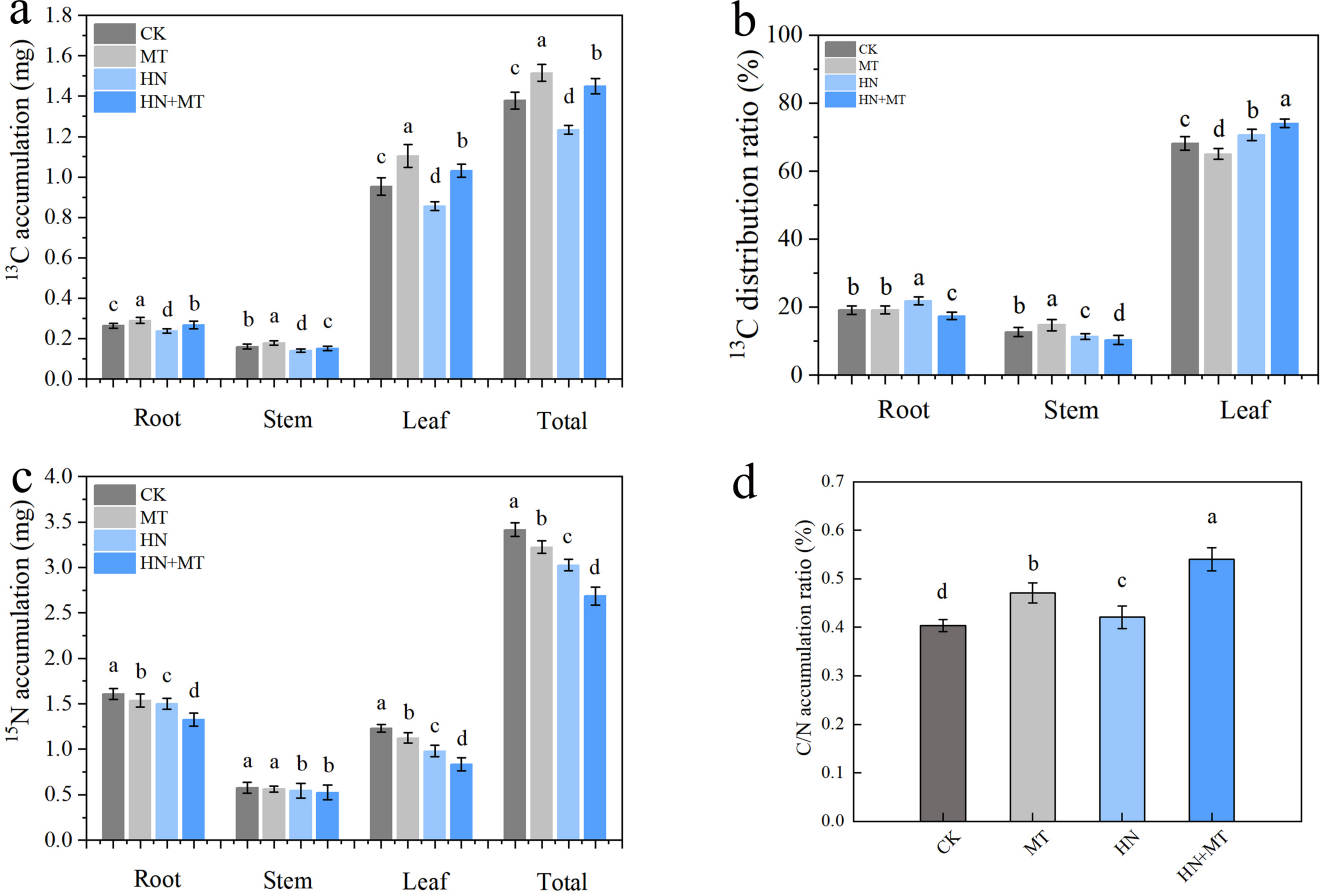
Effect of different treatments on ^13^C and ^15^N accumulation of M9T337 rootstocks. **(A)** ^13^C accumulation. **(B)** ^13^C distribution. **(C)** ^15^N accumulation. **(D)** C/N accumulation. The error bar represents the standard deviation of the mean (n=3). Different lowercase letters indicate significant differences among treatments (Duncan test, *p* < 0.05).

Under different treatments conditions, ^13^C accumulation was highest in all of the organs under the MT treatment, followed by the HN+MT and CK treatments. The least ^13^C accumulated in all of the organs and the entire plant under the HN treatment (**Figure 6A**). Compared with HN treatment, the root, stem, leave and total ^13^C accumulation of HN+MT treatment were 23.85%, 7.63%, 20.44% and 17.53% higher, respectively (**Figure 6A**). The distribution rate of ^13^C was mainly concentrated in the root of HN treatment, the stem of MT treatment and the leaf of HN+MT treatment (**Figure 6B**). The ^15^N accumulation of CK and MT treatment was significantly higher than that of HN and HN+MT treatment. Compared with HN treatment, root, stem, leaf and total N accumulation of HN+MT treatment were 11.45%, 3.52%, 14.98% and 11.17% lower, respectively (**Figure 6C**). It can be seen from **Figure 6D** that the C/N accumulation rate of HN+MT treatment and MT treatment is higher than that of CK and HN treatment. Compared with HN treatment, the C/N accumulation rate of HN+MT treatment was 32.55% higher.

### 3.7 Correlation between different indicators of M9T337 rootstocks

**Figure 7:**
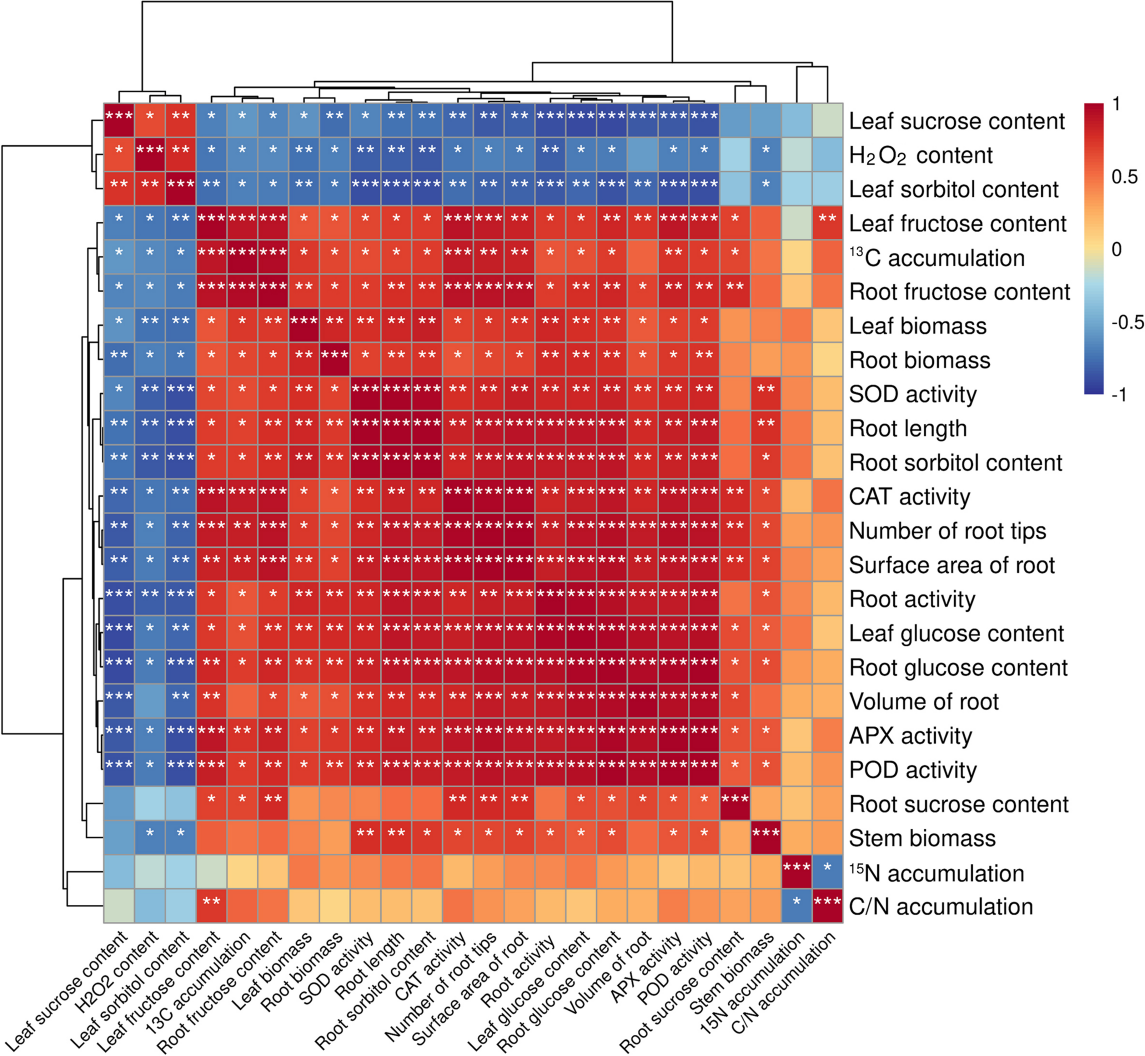
Correlation between different indicators of M9T337 rootstocks. (Duncan test, *p ≤ 0.05, **p ≤ 0.01, ***p ≤ 0.001).

In order to study the effects of different treatments on the rootstock of seedling M9T337, correlation analysis was carried out on its growth conditions, antioxidant protective enzymes (SOD, POD, CAT, APX) activity, soluble sugar content, carbon and N accumulation and distribution. As can be seen from **Figure 7**, leaf sucrose content was significantly negatively correlated with root activity, leaf glucose content, root glucose content, volume of root, APX activity and POD activity. The was negatively correlated with SOD activity, root sorbitol content, root length and root activity. Leaf fructose content was positively correlated with ^13^C accumulation, root fructose content, CAT activity, number of root tips, APX activity and POD activity. Among them, ^13^C and ^15^N are negatively correlated.

## 4 Discussion

### 4.1 Melatonin alleviated the inhibition of high N on the growth of rootstock

The major way that crops take in water and nutrients from the soil is through their roots. Proper root shape and structure are critical for plant growth and development as well as for the uptake of nutrients from the soil (Mulaudzi et al., 2020). Plants frequently allocate biomass preferentially to the roots in order to increase their capacity for nutrient uptake in response to nutritional deficiencies. For instance, low P stress encourages lateral root growth and increases the number of root hairs, while low N stress induces root elongation (Hu et al., 2024). The findings indicated that melatonin may successfully reduce the negative effects of excessive N on root development and vitality. The use of melatonin may effectively ameliorate the reduction of root length, root spots, root surface area, and root volume caused by high N pressure. Properly increasing the melatonin supply helps optimize the form and structure of roots in high-N environments by maximizing the absorption of N, which in turn encourages root development (**Figures 1A–F**). We conducted further measurements of the apple rootstocks’ biological mass. Our findings indicate that the application of high levels of N hindered plant development. Moreover, the addition of melatonin successfully mitigated the inhibitory effects of high levels of nitrate on plant growth (**Figures 1G–I**). These findings are concordant to the results of Zhang et al. (2017).

### 4.2 Melatonin reduced the oxidative damage of apple rootstock roots under high N

We also identified the antioxidant system’s metabolites and associated enzyme activity in the rootstock roots. The ROS equilibrium is maintained by plants by means of their inherent antioxidant systems (Boaretto et al., 2020). Among them, APX, CAT, POD, and SOD are crucial for sustaining and scavenging ROS (Choudhury et al., 2017). Under normal development circumstances, antioxidant protection enzyme activity is in a balanced state, but under high N suppression conditions, SOD activity, CAT activity and APX activity falls, weakening the function of root antioxidants. It lowers the efficiency of H_2_O_2_ clearance, promoting membrane lipid peroxidation, finally leading to root oxidation damage. This explains why excessive nitrate on produces greater oxidative damage to the root (**Figures 2E–I**). However, melatonin can significantly alleviate the peroxide damage caused by high N, and is conducive to removing hydrogen peroxide and reducing lipid peroxidation.

### 4.3 Melatonin increased carbon metabolism in M9T337 rootstock under high N stress

Plants convert CO_2_ dioxide into carbohydrates via photosynthesis, and these carbohydrates account for 90% of the biomass and agricultural productivity (Simkin et al., 2019). The carbohydrates essential for root development are transferred from the leaf, which shows that carbon partitioning is also a significant factor controlling root growth (Lambers et al., 2002). Some studies have shown that improving nitrogen (N) efficiency can lead to increased photosynthetic rates (Cechin and Valquilha, 2019). However, there is also evidence suggesting competition between nitrogen and carbon metabolism under high nitrogen conditions (Bloom, 2015). Our research indicates that under high nitrogen supply, the Pn, Gs, and Tr of apple rootstocks significantly decreased, while the addition of melatonin significantly enhanced photosynthetic capacity.

The effect of N in root growth promotion indicated via connections with carbohydrate content and metabolism (Li et al., 2018). In apples and certain other Rosaceae species, sorbitol and sucrose are the principal end-products of photosynthesis and are the main types of carbohydrates carried across large distances in the phloem (Sha et al., 2020). The findings revealed that high amounts of N compulsion and melatonin had a substantial influence on the accumulation and distribution of soluble sugar in M9T337 rootstock. Research has found that N fertilizer and melatonin have significant effects on plant carbon metabolism (Yan et al., 2023). In the absence of an increase in melatonin, high N coercion raised the level of peanut and mercury in the leaves and dramatically decreased the amounts of root-based peanut alcohol and mercury, showing that high nitrous coertion prevented the transfer of leaves from leaves to roots. This is comparable to the results of the research on apple (*Malus hupehensis*) (Zhao et al., 2020). Our results also show that high N stress limits the transport of photosynthetic products, as sorbitol content in leaves increases while sorbitol content and sucrose content in roots decreases. Increasing the supply of melatonin helps to alleviate the restriction of high N on the transport of photosynthetic products (**Figure 3**). Sorbitol and sucrose, as photosynthetic products can be degraded to fructose and glucose by SDH and SuSy after unloading to the root via long-distance transport in the phloem (Zhao et al., 2020). High N coercion reduces the content of fructose and glucose in the root. High nitrous coertion may reduce the activity of the related enzymes, resulting in a decrease in the concentration of frucose and glycine in the roots, thereby affecting the cycle of triglyceride and starch synthesis, inhibiting root growth (Li et al., 2012). Further analysis shows that the addition of melatonin in high N conditions promotes the phosphoridation of the fructose and glucose, thus enhancing the absorption and utilization of M9T337 rootstocks.

To further investigate the accumulation and distribution of photosynthetic products, M9T337 rootstock was tagged with ^13^C isotope. We found that the accumulation of ^13^C in leaves, stems and roots was the highest with melatonin administration, whereas the accumulation of ^13^C in roots under high N treatment was the lowest. High N treatment hindered N buildup and lowered C/N accumulation rate. Therefore, high N treatment inhibited the transport of photosynthetic products to roots, while appropriately increasing the melatonin supply level could increase and promote the distribution of sorbitol and sucrose from leaves to roots and increase the inhibition, thus reducing the inhibition of high N treatment on root growth.

### 4.4 Melatonin alleviated the inhibition of N assimilation in M9T337 rootstocks under high N stress

The application of N is frequently used in excess to achieve increased crop yields (Sun et al., 2020). Nevertheless, this study discovered that an overabundance of nitrate supply hindered the growth of the M9T337 apple rootstocks’ roots and decreased the ratio of roots to shoots, which was unfavorable for the absorption of nutrients and the growth of the rootstocks. After nitrate is absorbed and transported by the roots, it is reduced to nitrite, glutamine, and glutamate in sequence under the action of NR, GS, and GOGAT, and then further metabolized into amino acids and nitrogen-containing compounds. We quantified the absorption of NO_3_^−^ with the use of non-invasive micro measuring techniques. The net fluxes of NO_3_^−^ at the root surface was effluxed under circumstances of elevated nitrate levels, suggesting that high N concentrations hindered the absorption of NO_3_^−^. This is due to when the intracellular N levels are too high, key enzymes in the metabolic pathways are inhibited to prevent further accumulation of N. N sensors, such as TARGET OF RAPAMYCIN (TOR) signaling pathway, are activated to reduce N uptake and utilization (Artins et al., 2024). This mechanism helps cells maintain N balance and avoid toxicity from excess N. It has been reported that genes encoding enzymes related to N assimilation are regulated by carbon and N interactions (Guti´errez et al., 2007). Our findings revealed that under circumstances of high N levels, the activities and expression of NR, GS, and GOGAT were dramatically suppressed, while melatonin can significantly relieve the symptoms, which may be related to the fact that melatonin changed the C/N ratio of seedlings.

The absorption of nutrients in plants is regulated by both internal signals inside the plant and external signals from the environment. When the intensity of nutrient supply surpasses the concentration needed for the plant to develop at its maximum rate, the uptake of nutrients is primarily controlled by the intensity of the nutrient pool. Nitrate uptake is mostly attributed to *NRTs* (Wang et al., 2012; Yuan et al., 2022). *MdNRT1.1*, *MdNRT1.5* and *MdNRT2.1* are significant *NRTs* that are predominantly engaged in NO_3_^−^ absorption in the roots (Xuan et al., 2017). We observed that the expression levels of *NRT1.1*, *NRT1.2* and *NRT2.1* in the roots fell dramatically under high N conditions, which explained why high N stress hindered N uptake. However, it is not obvious if the reduced expression of these genes is regulated by internal or external N levels, and its precise mechanism requires additional exploration.

Excessive N application hampers the growth of M9T337 rootstocks by decreasing root-to-shoot ratios and nutrient absorption. High nitrate levels inhibit NO_3_^−^ uptake, activate N sensors like the TOR pathway, suppress key N metabolism enzymes, and inhibit the synthesis of soluble proteins, while melatonin can alleviate these effects. Additionally, the expression of vital nitrate transporters decreases under high N conditions, highlighting the intricate relationship between nitrogen levels and nutrient absorption, with melatonin serving as a potential mitigator of high N stress.

## 5 Conclusion

**Figure 8:**
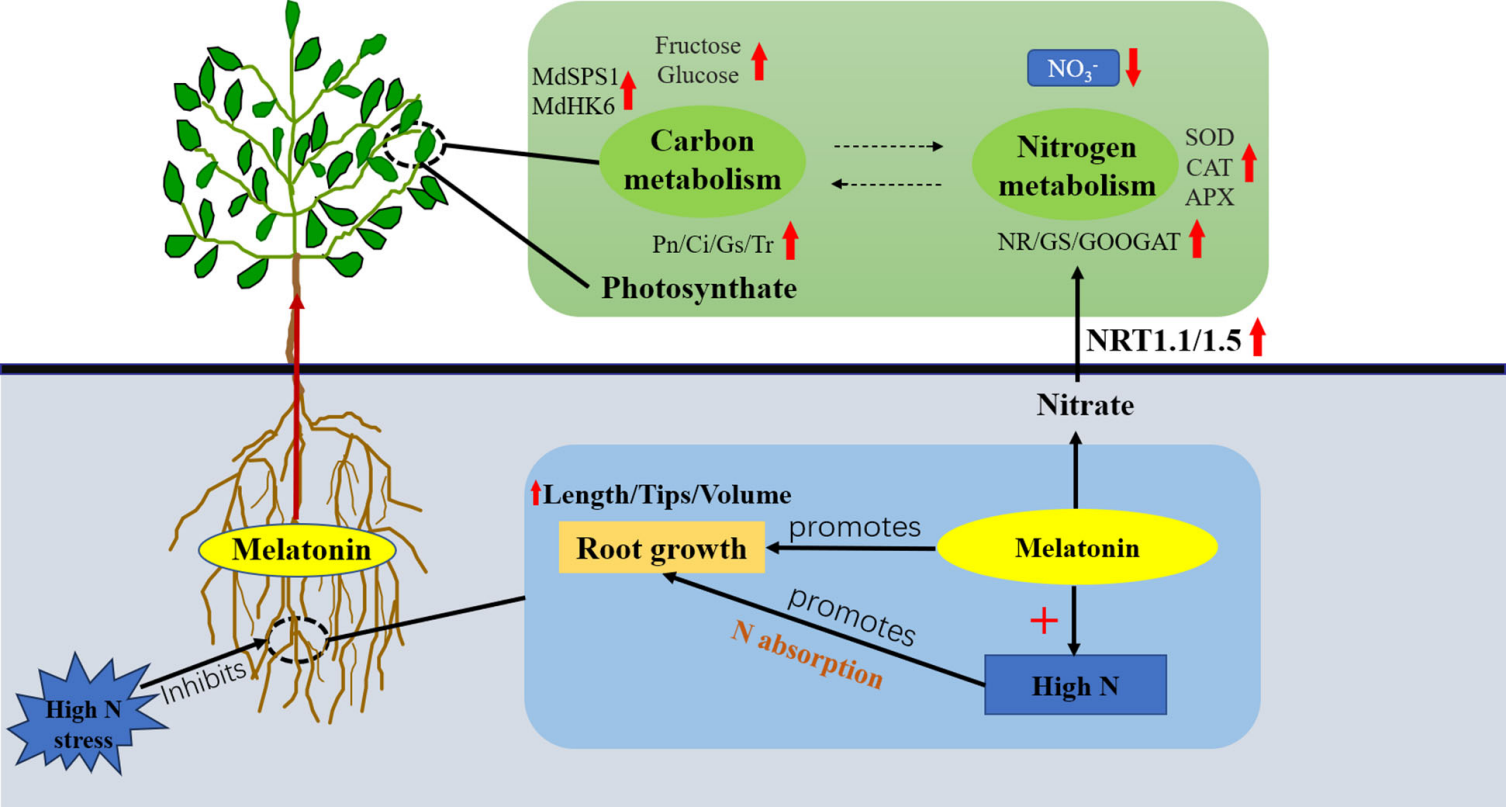
Model of the effect of exogenous melatonin on carbon and N metabolism in apple rootstock under high N stress.

In conclusion, correctly raising the melatonin supply level is helpful to root development of M9T337 rootstock under severe N stress. Under high N conditions, appropriately increasing melatonin level can activate antioxidant enzyme activity, reduce lipid peroxidation in roots, protect root structural integrity, promote the transport of sorbitol and sucrose to roots, and promote further degradation and utilization of sorbitol and sucrose in roots, which is conducive to the accumulation of photosynthetic products. At the same time, MT can also improve N metabolism, promote NO_3_^−^ uptake by roots, and raise the buildup of ^15^N (**Figure 8**). In conclusion, these results offer new insights for optimizing N application and maintaining carbon-nitrogen balance, which can contribute to improving apple yield and quality.

## Data Availability

The original contributions presented in the study are included in the article/**Supplementary Material**. Further inquiries can be directed to the corresponding authors.
