# Supplementary material for: Melatonin mitigates root growth inhibition and carbon-nitrogen metabolism imbalance in apple rootstock M9T337 under high nitrogen stress
Source: Front Plant Sci. 2024 Oct 14;15:1482351. doi: 10.3389/fpls.2024.1482351 (PMC11513380; doi:10.3389/fpls.2024.1482351)
Supplement: Supplementary file 1 [file Table1.docx]

**Table S1** Primer sequences for qRT-PCR.

| Gene  name | Forward sequence of the primers  (5′→3′) | Reverse sequence of the primers  (5′→3′) |
| --- | --- | --- |
| *MdNRT1.1* | CTCGGCCTCATTGTGTTCTT | TCCAACGGCAGTTCCATATTC |
| *MdNRT1.2* | TTAATTGCTGCCACACTTCATAG | CACGATGTTTGGTTCTGATACTTC |
| *MdNRT2.1* | GCTGTACTCTTCCTGTGACTTT | CGTCGACTTCTCGACATCTTT |
| *MdNRT1.5* | CGGAGGATACCAACCAAACA | GTTCATGGCCAGGTAGAAGTAG |
| *MdNR* | GTCACACGAGTGGAGATAACAA | CAGAAACACCAGCACCAGTA |
| *MdGS1* | ATATCTGCTGGAGATGAACTGTGG | TGGACTTGGTGCTGTAGTTTGTG |
| *MdNADH-GOGAT* | ACTATGGTCGGTTCTCAAC | TCTTGATGCCTCTTGCTAA |
| *MdSUSY1* | CTC AAG CGT GTT AAG CAA CAG | CTG AAT GGA ACA CGA AGA ATA TC |
| *MdSUT1* | TGT TCC GTA TGC TTT GGT TTC TTC | AAT AGC TGA TCC CAA GGT CCA CT |
| *MdSPS1* | AGT GTA GTA CTC AAG GGA GTT GG | TGC TCA TGG GGA AGG CTT TAC |
| *MdHK6* | GTG GGG CAG AGT GTT TGG TGT T | AAC CAC CGT CAG AGG CCA AAC C |
| *MdActin* | CATGGTTGGTATGGGTCAGAAG | GTCATCCCAGTTGCTCACTATG |
